# The parable of the Therapeutic Goods Administration approval of esketamine (Spravato) in Australia

**DOI:** 10.1177/10398562231156475

**Published:** 2023-02-20

**Authors:** William Lugg

**Affiliations:** Department of Psychiatry, 7799 The Alfred Hospital, Melbourne, Australia

**Keywords:** ketamine, esketamine, Janssen-Cilag, therapeutic goods administration, advisory committee for medicines

## Abstract

**Objective:**

To review the sequence of events that led to the Therapeutic Goods Administration (TGA) approval of esketamine in Australia, and to explore the potential ethical and clinical consequences of it.

**Conclusions:**

Trust in the TGA is of paramount importance to Australian psychiatrists. The approval of esketamine raises serious questions about the processes, independence and authority of the TGA, and therefore the confidence Australian psychiatrists can have in the ‘quality, safety and efficacy’ of the drugs they offer their patients.

The Therapeutic Goods Association (TGA) is Australia’s national regulatory authority for ‘therapeutic goods’. The TGA claims to ‘carry out a range of assessment and monitoring activities to ensure therapeutic goods available in Australia are of an acceptable standard’.^1^ ‘Therapeutic goods’ include prescription medicines, such as esketamine. The *Therapeutic Goods Act 1989* (hereafter, the Act) requires that medicines imported into, or exported from, Australia must be included in the Australian Register of Therapeutic Goods (ARTG).^2^ In order for a prescription medicine to be included in the ARTG, a ‘sponsor’ (i.e. company or individual) is required to submit an application to the TGA. A submission to register a prescription medicine must include a) data to support the quality, safety and efficacy of the product for its intended use, b) completed forms and c) payment of fees. As part of the submission evaluation process, the TGA may seek advice from two expert committees: 1) the Advisory Committee on Prescription Medicines (ACPM) and, 2) the ACPM Pharmaceutical Subcommittee (PSC).^1^

In January 2019, Janssen-Cilag submitted an application to register Spravato (esketamine hydrochloride) under the ARTG for the proposed therapeutic indication:

*“Treatment resistant depression, defined as MDD in adults who have not responded adequately to at least two different antidepressants of adequate dose and duration, to treat the current depressive episode.”* ^3^

As part of their application, Janssen-Cilag provided four pivotal/main efficacy studies (described as ‘active-controlled’) and one open-label study (see Table 1); no study was labelled ‘placebo-controlled’. The TGA commented that none of the studies were actually ‘active-controlled’, however, as there was ‘no active comparator to the tested drug’ (i.e. intranasal esketamine).^3^

**Table 1.** Efficacy studies submitted by Janssen-Cilag.^3^

Pivotal/main induction		
Study	Description	Comments
TRD3001	Randomised, double blind, multicentre, active-controlled study to evaluate the efficacy, safety and tolerability of fixed doses of intranasal esketamine plus an oral antidepressant in adult subjects with treatment-resistant depression	- ‘None of the studies were actually active-controlled as there was no active comparator to the tested drug (that is, intranasal esketamine)’. (TGA, 2021) - ‘Only Study TRD3002 demonstrated statistical superiority of flexible dosing of intranasal esketamine + oral antidepressant over oral antidepressant + intranasal placebo treatment. The other two pivotal induction studies (including elderly subjects in Study TRD3005) were negative. No meta-analysis of the double blind induction studies was undertaken’. (TGA, 2021) - The one study (TRD3002) that did reach statistical significance (*p* = 0.034) included a change in MADRS total score of −19.6 (ketamine + oral AD) versus −16.3 (placebo + oral AD); a difference in total score of just 3.3 between esketamine and placebo
TRD3002	Randomised, double blind, multicentre, active-controlled study to evaluate the efficacy, safety and tolerability of flexible doses of intranasal esketamine plus an oral antidepressant in adult subjects with treatment-resistant depression	
TRD3005	Randomised, double blind, multicentre, active-controlled study to evaluate the efficacy, safety and tolerability of flexible doses of intranasal esketamine plus an oral antidepressant in elderly subjects with treatment-resistant depression	
TRD3003	Randomised withdrawal design, double blind, multicentre, phase III, active-controlled study of intranasal esketamine plus an oral antidepressant for relapse prevention in treatment-resistant depression	- ‘Actually placebo-controlled due to the lack of an active comparator to intranasal esketamine’. (TGA, 2021) - ‘Would have been a better designed maintenance study if the double blind studies, Studies TRD 3001 and TRD 3002, had adequate number of patients fulfilling both the stable remission response and stable non-remission response criteria for transferred entry into the maintenance study without contribution from the direct-entry route’. (TGA, 2021)
Open-label		
TRD3004	Open label, phase III, long-term, safety and efficacy study of intranasal esketamine in, treatment-resistant depression over 1 year duration	- Flexible dosing (thus raising questions about dose-related efficacy). - Unblinded, uncontrolled, non-randomised

In September 2019, the TGA’s clinical evaluator had completed the second of two initial evaluations and recommended against approval of the drug due to a ‘lack of efficacy data that supports the proposed indication’.^3^ In March 2020, The TGA sought expert advice from the Advisory Committee for Medicines (ACM) who ‘agreed that Spravato had an overall negative benefit-risk profile for the proposed indication’^3^ and declared there was “significant and unacceptable uncertainty associated with the claims of efficacy of esketamine in patients with Treatment Resistant Depression (TRD).”^3^ Instead of rejecting the application outright, however, Janssen-Cilag was ‘encouraged and given the opportunity by the TGA, to submit any new data it might hold on a properly designed clinical trial’.^3^ Janssen-Cilag responded by providing preliminary data from an incomplete, open-label trial and a re-analysis of data they had already submitted. The TGA did not consider this response “sufficient”, and noted that Janssen-Cilag had not provided ‘any new data from a properly designed clinical trial of adequate duration’.^3^ In September 2020, citing ‘irresolvable methodological issues’, the ACM rejected the application and concluded: (Table 2)

*“Spravato has an overall negative benefit-risk profile for the proposed indication, as the evidence submitted did not satisfactorily establish the efficacy of the product with certainty. There is also doubt about the practicality of the RMP [Risk Management Profile] to support the management of significant safety and toxicity concerns.”* ^3^

**Table 2.** Therapeutic Goods Association (TGA) timeline of events leading to the registration of esketamine (Spravato).^3^ (Available from TGA website)

Submission dossier accepted and first round evaluation commenced	2 Jan 2019
First round evaluation completed	11 Jul 2019
Sponsor provides responses on questions raised in first round evaluation	6 Aug 2019
Second round evaluation completed	12 Sep 2019
Delegate’s overall benefit-risk assessment and request for Advisory Committee advice	2 Mar 2020
Sponsor’s pre-Advisory Committee response	13 Mar 2020
Advisory Committee meeting	2 and 3 Apr 2020
	4 and 5 Jun 2020
Registration decision (outcome)	10 Sep 2020
Number of working days from submission dossier acceptance to registration decision*	188
Section 60 appeal decision (initial decision revoked)	27 Jan 2021
Registration decision (section 60 - approval)	5 Mar 2021
Completion of administrative activities and registration on ARTG	9 Mar 2021

*Statutory timeframe for standard applications is 255 working days.

On the 27th January 2021, Janssen-Cilag appealed this decision under Section 60 of the Act*,* which states that ‘a person whose interests are affected by an “initial decision” made under that Act, may request the [Health Minister] to reconsider the initial decision’.^4^ Under the Act, ‘a person’ can apparently include a multinational drug company. In other words, understandably dissatisfied with the outcome of their application, Janssen-Cilag circumvented the inconvenient request to provide ‘a properly designed clinical trial’ and, lawfully, sought ministerial-level intervention. Just five weeks later (as opposed to the twenty-one months it took the TGA and ACM to reach their considered conclusions), Spravato was approved for registration under the ARTG at the behest of a delegate acting on behalf of the then Health Minister, Greg Hunt (hereafter, the Delegate).^3^

Scrutinised below are the Delegate’s key reasons for overruling the TGA and ACM:

1. The Delegate determined that even though statistically significant results were only seen in one of the submitted efficacy trials, the effect sizes in the other two trials were ‘similar’^3^ (thus, by implication, the results were considered a reasonable demonstration of efficacy). Such reasoning suggests that achieving statistical significance is relatively unimportant. Failure to achieve statistical significance actually suggests that the results could have occurred purely by chance and that the drug was no better than comparator.
2. The Delegate determined that adverse effects were ‘generally non-serious in nature’ and that such effects could be ‘managed by appropriate post-dose observation’. Dose? According to Janssen-Cilag^5^’s own data, the inter-subject variability of absorption of esketamine through nasal muscosa (measured in C_max_) ranges from as little as one-quarter of the dose to as much as two-thirds. Given adverse effects of ketamine have been reported elsewhere as dose-related,^6^ it follows that such variability in dose-absorption will manifest in variability in nature and severity of adverse effects. The most common reported adverse effect of esketamine is dissociation^5^; to declare this ‘non-serious’ is highly contentious. Some evidence suggests ketamine-induced dissociation can be highly traumatic with potentially serious and prolonged consequences.^7^ Ketamine-related dissociation has been associated with higher doses and faster absorption^6^ – potentially variable parameters with Spravato. Thus, it is difficult to predict who will suffer dissociation and just how badly, let alone the longer-term consequences of it. Finally, the incidence rate of ‘suicidality-related events’ with esketamine was twice that of placebo (with three completed suicides on esketamine, none on placebo).^3^ Given all this, the ACM’s doubts about the ‘practicality of the RMP to support significant safety and toxicity concerns’^3^ were entirely apt.
3. The Delegate appeared to acknowledge the TGA’s concerns about the methodology of the pivotal induction efficacy studies but ostensibly disregarded those concerns because they were deemed ‘acceptable to comparable overseas regulators’,^3^ including the US Food and Drug Administration (FDA). The ‘argument from authority’ (i.e. that because the FDA accepted these methodologies, the TGA should) is not a valid, nor appropriate, argument for an independent national regulator. Furthermore, if the opinion of international regulatory bodies *are* worth referencing, then it is noteworthy the Delegate neglected to mention that of the UK National Institute for Health and Care Excellence (NICE). In May 2022, NICE, for a third time, rejected Spravato on the grounds of inadequate clinical and cost-effectiveness.^8^
4. The Delegate commented that Janssen-Cilag had provided ‘expert opinion’ that commencing esketamine with a new oral antidepressant ‘reflected optimal clinical practice’^3^ (and, by implication, this made the submitted trials that used that design acceptable). Who exactly gave the ‘expert opinion’ is not disclosed, but the problems with this ‘opinion’ are obvious. By initiating a ‘new’ (i.e. novel to the person) antidepressant in conjunction with esketamine, the efficacy of either drug is confounded, irrespective of whether the person had previously ‘responded’ to other antidepressants or not. As the TGA and ACM correctly pointed out, efficacy of esketamine over placebo cannot reliably be established with this design. The ‘expert opinion’ of a multinational drug company (on one of their own products) does not outweigh the fundamentals of critical appraisal.
5. The Delegate accepted the assertion (made by whom is undisclosed) that it would be ‘unethical’ for a patient with treatment-resistant depression (TRD) to be ‘left without any antidepressant treatment’^3^ (i.e. patients receiving placebo in a hypothetical placebo-controlled esketamine, monotherapy study for TRD). This is a contentious assertion that assumes drug therapeuticity in the *absence* of a monotherapy placebo-controlled study – a dangerous assumption. Even if an experimental neurochemical, like esketamine, did have putative ‘antidepressant properties’, it is arguably more unethical to offer this to people clinically (i.e. not experimentally) *without* sufficiently determining superiority over placebo.

Despite these issues the Delegate overruled the national drug regulator and, on the 5^th^ March 2021, the TGA officially approved the registration of Spravato with a revised indication and a requirement for a new oral antidepressant to be commenced simultaneously:

Spravato is indicated for treatment resistant depression (Major Depressive Disorder in adults who have not responded adequately to at least two different antidepressants of adequate dose and duration to treat the current moderate to severe depressive episode).

Spravato is to be initiated in conjunction with a newly initiated oral antidepressant.^3^

If the Delegate’s reasons for overturning the (repeated) negative evaluations of the TGA and ACM seem difficult to fully understand (even if just from a scientific point of view), it is worth considering other *potentially* influential factors. It is noteworthy that between 2016 and 2020, Janssen-Cilag donated $166,955 to the Liberal/Nationals – the known sum, the vast majority (64.9%) of donations came from undisclosed sources.^9^ Similarly, between 2016 and 2018, the Labor party received $197,400 from Janssen-Cilag, with 55% of their donation sources remaining undisclosed.^9^ Such corporate donations are not uncommon and, in themselves, do not amount to evidence of corruption. They do, however, raise questions of influence. The reader can speculate as to why a multinational drug company would donate such vast sums of money to Australian political parties who, when in government, oversee the organisation responsible for evaluating their products. In the interests of ensuring the independence and integrity of our national drug regulator, perhaps such (pharma-derived) donations should hereon be prohibited or, at the very least, publicly disclosed, real-time and made immediately visible on the TGA’s information page for any product manufactured by the donor.

The story behind the TGA approval of Spravato is of utmost importance to Australian psychiatrists. Australian psychiatrists rely on the TGA to safely regulate drugs (and other therapeutics) in a way that is scientifically, procedurally and ethically sound. Drugs that are approved by the TGA are used by psychiatrists to try and help people suffering from severe and diverse forms of mental illness. Sufferers, in turn, rely on psychiatrists to offer them drugs they trust have been approved for use based on a thorough and independent evaluation of their ‘quality, safety and efficacy’. The trust bound within these relationships cannot be overstated. Esketamine repeatedly failed the TGA and ACM’s independent evaluations, only to be swiftly overturned by the Minister’s office following an appeal from the manufacturer. It is extraordinary that whilst the Delegate thought it ‘unethical’ for a person suffering TRD to go ‘without an antidepressant’ (i.e. esketamine),^3^ it was deemed ethical for the Delegate to overrule the repeated negative assessments of the independent and impartial national drug regulator *and* its’ separate drug advisory committee.

This story is not about Big Pharma bashing, nor is it an accusation of corruption. Janssen-Cilag did, legally, what any profit-seeking multinational corporation would do. It is neither a dismissal of the possibility that esketamine may be useful to some people, in some circumstances, some of the time.^10^ It is, however, a disconcerting narrative that raises questions about the methods, integrity and authority of the national drug regulator. If psychiatrists lack confidence in the processes and judgements through which drugs are approved for use in this country, how can they have confidence that the drugs they offer their patients are of truly adequate ‘quality, safety and efficacy’?
